# Methods to Quantify the Importance of Parameters for Model Updating and Distributional Adaptation

**DOI:** 10.1177/0272989X241262037

**Published:** 2024-07-26

**Authors:** David Glynn, Susan Griffin, Nils Gutacker, Simon Walker

**Affiliations:** Centre for Health Economics, University of York, York, UK; Centre for Health Economics, University of York, York, UK; Centre for Health Economics, University of York, York, UK; Centre for Health Economics, University of York, York, UK

**Keywords:** sensitivity analysis, value of information, distributional cost effectivness analysis

## Abstract

**Purpose:**

Decision models are time-consuming to develop; therefore, adapting previously developed models for new purposes may be advantageous. We provide methods to prioritize efforts to 1) update parameter values in existing models and 2) adapt existing models for distributional cost-effectiveness analysis (DCEA).

**Methods:**

Methods exist to assess the influence of different input parameters on the results of a decision models, including value of information (VOI) and 1-way sensitivity analysis (OWSA). We apply 1) VOI to prioritize searches for additional information to update parameter values and 2) OWSA to prioritize searches for parameters that may vary by socioeconomic characteristics. We highlight the assumptions required and propose metrics that quantify the extent to which parameters in a model have been updated or adapted. We provide R code to quickly carry out the analysis given inputs from a probabilistic sensitivity analysis (PSA) and demonstrate our methods using an oncology case study.

**Results:**

In our case study, updating 2 of 21 probabilistic model parameters addressed 71.5% of the total VOI and updating 3 addressed approximately 100% of the uncertainty. Our proposed approach suggests that these are the 3 parameters that should be prioritized. For model adaptation for DCEA, 46.3% of the total OWSA variation came from a single parameter, while the top 10 input parameters were found to account for more than 95% of the total variation, suggesting efforts should be aimed toward these.

**Conclusions:**

These methods offer a systematic approach to guide research efforts in updating models with new data or adapting models to undertake DCEA. The case study demonstrated only very small gains from updating more than 3 parameters or adapting more than 10 parameters.

**Highlights:**

- It can require considerable analyst time to search for evidence to update a model or to adapt a model to take account of equity concerns.
- In this article, we provide a quantitative method to prioritze parameters to 1) update existing models to reflect potential new evidence and 2) adapt existing models to estimate distributional outcomes.
- We define metrics that quantify the extent to which the parameters in a model have been updated or adapted.
- We provide R code that can quickly rank parameter importance and calculate quality metrics using only the results of a standard probabilistic sensitivity analysis.

Decision models are used in health care to predict the consequences of a decision (e.g., to invest in a new health care technology or implement a specific policy) in terms of its overall impact on population health, taking account of both the benefits and opportunity costs. As increasing policy attention is paid to avoidable or modifiable inequalities in health between population groups, there is interest in estimating the distributional health consequences of decisions using distributional cost-effectiveness analysis.^1,2^ Further, when new evidence becomes available or as time passes, decisions on health care may need to be revisited.

De novo decision models are costly and time-consuming to build and validate. Therefore, updating existing model parameters to reflect new data or adapting existing models to capture the distribution of outcomes across equity relevant subgroups may represent a more efficient use of research resources. In this article, model “updating” will be used to refer to updating parameter values to incorporate evidence that has emerged since the original model construction, and model “adapting” will refer to converting an existing model to estimate a distribution of health consequences across equity relevant subgroups, by allowing parameter values in the model to vary across subgroups.

In the ideal case, all model inputs would be updated or adapted following best practice for evidence identification for each parameter (e.g., systematic review and evidence synthesis for treatment effectiveness). However, it is not practical to do this as decision models may contain hundreds of parameters and evidence identification or generation is a resource-intensive activity, ranging from simple searching, to secondary data analysis,^3^ to the commissioning of expensive primary research or expect elicitation.^4–6^

In some cases, for example, where a new major trial has been published, it may be obvious that a particular parameter should be updated. However, even in such cases it may not be apparent whether other parameters in the model require updating. In many cases there may be no prominent change in evidence, and the analyst will be required to invest time in ascertaining its presence. Given the resource demands of identifying evidence, it may be beneficial to rank parameters by their potential importance when considering updating or adapting models. In this article, we apply value of information (VOI) and 1-way sensitivity analysis in a novel way to aid parameter prioritization for 1) model updating and 2) model adaptation. Model updating and adaptation are discussed separately as different methods are appropriate for each application. Further, we propose metrics that can be used to assess the extent of the model update/adaptation.

We first provide an overview of methods used to assess parameter influence in decision models. Second, we propose how VOI and 1-way sensitivity analysis can be used to quantify parameter importance in model updating and adaptation, respectively. Third, we demonstrate our proposed methods using a breast cancer model. R code to quickly carry out the analysis is provided in the appendix.

## Methods to Assess Parameter Influence

Several tools have been developed to assess parameter influence in economic models.^7–9^ Broadly, the “influence” of a parameter refers to the degree to which the model results depend on the value of the parameter. For it to have practical importance, the concept of influence must also link to decision making. Hence, the relevant outcome is the impact on cost-effectiveness. Here, we use the incremental net benefit (INB), computed as shown below to capture the influence of parameters on the outcome of interest^7^:

INB=ΔQALYs−ΔCosts/k.

INB is the gain in quality-adjusted life-years (QALYs) from the intervention (ΔQALYs), minus the QALYs displaced by any additional costs of the intervention (ΔCosts), where k represents the rate at which QALYs are displaced by costs (i.e., the marginal productivity of the health system).^10^ Alternatively, k may be considered to represent the “willingness to pay” for an additional QALY.

### One-way sensitivity analysis

For OWSA, a parameter of interest is set to specific values to understand its impact on INB. The other parameters in the model may either be kept at their expected values or their joint uncertainty may be propagated to get an unbiased estimate of expected INB conditional on the value of the parameter of interest.^9,11^ The influence of the parameter of interest is quantified as the change in INB resulting from the change in the parameter of interest from its expected value. One-way sensitivity analysis (OWSA) isolates the impact of a parameter taking a particular value; it does not take account of the probability that the parameter will take that particular value. This has been described as a limitation of OWSA, but it also can be useful as it distinguishes the importance of a parameter in terms of capacity to influence outcomes from any uncertainty about the expected value of that parameter.^9,12^

#### Probabilistic sensitivity analysis and analysis of covariance

To take account of the probability of parameters taking specific values and the implications for INB, probabilistic sensitivity analysis (PSA) was developed. This calculates the variation in INB that results from the joint uncertainty in all parameters.^7^ Parameter uncertainty is typically expressed by parametric distributions whose spread captures uncertainty about their true value. Where possible, these distributions should reflect correlation between parameters. A PSA analysis results in a matrix of data that links specific input parameter values with predicted INB. Further processing is required to use the PSA to understand the influence of particular input parameters. Analysis of covariance (ANCOVA) methods have been proposed for this purpose.^7,8^ These methods use a statistical model to estimate the proportion of uncertainty in INB attributable to uncertainty in each input. Parameters that explain larger proportions of the overall variation in INB are considered to have more influence in the model. The proportion of variation explained by a parameter is determined by:

1. The degree to which the parameter influences INB in the decision model. Parameters that have a large influence explain a larger proportion of variance.
2. The degree of parameter variation in the PSA (i.e., its degree of uncertainty). All else equal, parameters with low variation are less likely to explain variation in INB.

Parameter influence is quantified as a proportion of overall variation, making it challenging to relate to decision making. However, ANCOVA can be useful to understand which outcomes may be driving PSA results.

#### Expected value of perfect information and partial perfect information

VOI methods estimate the value in health terms of resolving uncertainty in a particular parameter or set of parameters.^13–15^ These methods were primarily developed to estimate the value of funding additional research but have also been proposed as a method to assess the influence of specific parameters in a model.

The expected value of perfect information (EVPI) calculates the total value of resolving sampling uncertainty in every model parameter.^7^ It therefore provides an upper bound for the value of additional research based on the characterized uncertainty, although some uncertainty may not be reflected. However, it does not decompose this total value into the value of resolving uncertainty in individual parameters or sets of parameters. The expected value of partial perfect information (EVPPI) provides this information.^14^ EVPPI provides an upper bound for the value of resolving all uncertainty for a given parameter or set of parameters. The EVPPI for a parameter is determined by the following:

1. The degree of uncertainty about a parameter’s true value (i.e., the spread of its prior distribution). Parameters with greater uncertainty are more likely to have a large EVPPI.
2. The degree to which the parameter influences INB. Parameters that have a large influence on INB are more likely to have a large EVPPI.
3. The degree of decision uncertainty (i.e., how close the choice is between the treatment alternatives). When expected INB with current information is close to zero, it is more likely that EVPPI values will be large.

As noted previously, EVPPI can be calculated for a single parameter or a set of parameters. It should be noted that the sum of EVPPI for 2 or more parameters considered independently will not generally equal the EVPPI for the set of those parameters.^16^ The value of reducing uncertainty in a given parameter is conditional on the joint uncertainty in the other parameters.^16^ Therefore, to calculate the EVPPI for research that gathers information on a set of parameters, the EVPPI should be calculated for the full set of parameters on which evidence would be collected. The judgment about which parameters should be considered independently and which should be analyzed as a set will depend on the specific application and decision-making context.

In the literature, EVPPI has primarily been articulated as an approach to estimate benefits of funding additional primary research on a given parameter or set of parameters.^7,14^ This application has a clear link to decision making through research-funding decisions. However, it generalizes to any costly effort to reduce parameter uncertainty, including undertaking a literature review. It has also been described as a method to understand parameter influence in a decision model, where parameters with larger EVPPI are considered to have greater influence when analyzing decision uncertainty.^8^

## Methods

This section describes the proposed methods and interpretation required to prioritize parameters to 1) update an existing decision model with new data and 2) adapt a decision model to reflect differences between population groups. R code to carry out the analysis is provided in the appendix.

Throughout, we assume that the original model was fit for purpose at the time of being used (i.e., it provided an unbiased estimate of INB). In cases in which this assumption does not hold, the proposed methods are unlikely to be suitable, and a new evaluation and model may be required.

It is necessary to make the distinction between ex ante and ex post. Ex ante refers to the state of knowledge before new efforts to gather information on the parameters of interest. Ex post refers to the state of knowledge after searching. To be useful for prioritization, the methods proposed must be feasible ex ante. This means they must be based on information available to the analyst before searching the literature (i.e., they must be based on the decision model only).

### Prioritizing Parameters to Update a Standard Decision Model with New Data

The goal in updating a decision model is to incorporate more precise, up-to-date information on input parameters (i.e., use new data to reduce uncertainty in the parameter inputs). Those inputs with higher value of uncertainty may be considered the most important for updating.

OWSA does not take account of the joint uncertainty in parameter values. Further, OWSA based on the 95% interval does not capture the full distribution of uncertainty in parameter values.^9^ ANCOVA may provide a useful quantification of the relationship between parameter uncertainty and outcome uncertainty; however, it does not link directly to decision uncertainty. EVPPI methods provide a well-established and well-described framework for quantifying the value of reducing decision uncertainty in parameters.^7,13,17^ EVPPI has also been proposed as a method to inform systematic reviews.^18^ In this section, we build on these previous applications and propose methods that use EVPPI to rank parameter importance and construct an update metric.

#### Ranking model importance by EVPPI

We first estimate EVPPI for each input parameter (or set of parameters) independently. In the presence of a large number of parameters, efficient methods to calculate EVPPI may be required.^14,19^

The “independent EVPPI” calculation can provide a pragmatic first approximation of the order in which to prioritize parameters for updating. However, as the EVPPI for a given parameter is conditional on the joint uncertainty in the other parameters (i.e., reflecting the correlations), additional steps are required to calculate an appropriate ranking that takes this into account; these are detailed below.^16^

The parameter with the highest EVPPI is ranked first. However, the parameter with the second highest independent EVPPI will not necessarily be rank 2, as the learning about some parameters might reduce or increase the value of learning about others. Therefore, after the rank 1 parameter has been identified, EVPPI for each remaining parameter must be recalculated conditional on knowing the rank 1 parameter. The parameter, which in combination with the rank 1 parameter, has the largest EVPPI is rank 2. EVPPI must then be calculated conditional on knowing the rank 1 and 2 parameters, and so on. This is continued until the gain in EVPPI from adding additional parameters goes to zero or all parameters have been included. See the algorithm in Box 1.

**Box 1:** Algorithm for Calculating Conditional EVPPI to Rank a Set of Parameters

**Conditional EVPPI for ranking parameter priority** 1. Calculate EVPPI for all parameters independently. 2. List parameters by size of EVPPI; the parameter with the highest EVPPI is ranked 1. 3. Calculate the EVPPI for the remaining parameters combined with the currently ranked parameter(s). 4. From the analysis in step 3, list the parameters by size of EVPPI; the largest is the next parameter in the ranking. 5. Repeat steps 3 and 4 until all parameters have been ranked or marginal increases in EVPPI have gone to zero.

EVPPI, expected value of partial perfect information.

#### Calculating model update metric

EVPPI can also be used to construct an update metric that can be presented alongside a model update. This quantifies the extent to which the parameters that were updated in the model contributed to the overall decision uncertainty. This is calculated by computing the conditional EVPPI for the set of all parameters that have been updated, then dividing this by the EVPI. Because EVPI represents the value of reducing all parametric uncertainty in the model, this will theoretically equal 100% if all relevant parameters have been updated. This metric can be calculated for each step in the EVPPI ranking described above. This results in an illustration of the gain in update comprehensiveness from updating additional parameters with new information.

### Prioritizing Parameters to Adapt a Standard Decision Model to Become a Distributional Model

In the case of adapting a model to estimate the distribution of health consequences across populations, parameters within the model could again be prioritized on the basis of their impact on expected INB. The parameter prioritization task can therefore be informed by quantifying the INB impact of adapting the expected value for each of the parameters in the model. Naturally, some parameters may differ across groups more than others (i.e., some parameters may have steeper social gradients). However, knowledge of these differences may be available only ex post, and so it cannot be used in ex ante search prioritization.

The ranking provided by the EVPPI calculations in the model updating section is not appropriate for parameter prioritization for model adaptation. This is because the value of knowing an input parameter with certainty is not the same as knowing the impact that a change in the expected value of that input parameter would have on model outcomes.^9^ It is the latter that we argue is relevant in model adaptation. The use of ANCOVA in model adaptation suffers from similar issues as EVPPI. This is because it is again based around understanding the implications of uncertainty in input parameters rather than changes in expected value.

In this article, we propose OWSA as a feasible method to prioritize parameter searching and construct an adaptation metric ex ante. The argument for this method is that it allows for consideration of the impact of changes in parameter expected value, separate from considerations of uncertainty in that parameter. OWSA provides an estimate of the degree to which the parameter influences INB. Parameters that have a large influence on outcomes are more likely to influence full population outcomes and between-group differences. Those parameters with larger influence should be prioritized in model adaptation.

#### Rank model importance by OWSA

To calculate the importance of each parameter, the first step is to calculate the impact of a common, arbitrary (e.g., 1%) increase in each input parameter on INB (i.e., calculate a semielasticity for each parameter).

OWSA can be carried out manually, by one-at-a-time changing parameter values by 1%, calculating change in INB and recording results. Alternatively, for an approximately linear model, meta-modeling based on ordinary least squares can be used to quickly calculate semielasticities based on information produced by the PSA.^12^ To achieve this, a linear model is fitted with INB as the dependent variable and all model inputs as independent variables, where the independent variables are rescaled to be percentage differences from their mean. The 1% semielasticities are given by the estimated coefficient values. The second step is to rank every parameter in order of largest absolute semielasticity.

#### Calculating model adaptation metric

To calculate the adaptation metric, normalize the absolute semielasticities to 100% by dividing each absolute semielasticity by the sum of all absolute semielasticities. This gives the percentage of total variation covered by each input parameter. By summing the proportion modeled ex ante, an analyst can report an adaptation metric that provides decision makers with an estimate of the degree to which important parameters in the model have been adjusted to reflect equity relevant differences. This will equal 100% if all relevant parameters have been updated. Note this metric captures the impact of a common change in each parameter.

## Case Study

The example model is a Markov model of cancer with 4 states: well, local recurrence, metastasis, and dead. This model compared treatment A (current practice) to treatment B for a UK population. This model is loosely based on a real model but is used here for illustrative purposes only. Treatment B was both higher intensity (i.e., a higher number of sessions) and used an innovative modality (focused on a section of the breast rather than the full breast). Model structure and inputs are reported in the appendix.

The model had 41 total parameters, 21 probabilistic and 22 deterministic. Deterministic inputs were starting age, discount rates, background mortality, treatment effect duration, change in utility with age, and components of intervention cost. The probability of short-term side effects with each treatment were the only input parameters that were correlated in the model with all other parameters being considered to be independent. Model survival functions were all exponential.^8,12^

After 10,000 PSA samples, treatment B was expected to dominate A providing 0.0226 additional QALYs and cost savings of £1,806 per person. There was little decision uncertainty, and B had a 99.5% probability of being more cost-effective based on a cost-effectiveness threshold of £13,000 per QALY.^10^ The incident population in the United Kingdom is 24,799 patients per year.

### Which Parameters Should Be Prioritized when Updating in a Standard Decision Model with New Data?

To estimate EVPPI for each input parameter (or set of parameters as appropriate) with minimal computation time, we used an efficient calculation method based on a generalized additive model.^14^ The EVPPI estimates are multiplied by the incident population for presentation purposes, although this is not required.

Only 2 parameters had nonzero EVPPI: the relative effect for treatment intensity (0.36 QALYs per year) and the relative effect for the new modality (0.19 QALYs) on local recurrence. According to this “independent EVPPI” ranking, the relative effect for treatment intensity is highest priority for updating.

The EVPPI for updating both parameters simultaneously is 2.41 QALYs, which differs from the sum of independent EVPPI values, as expected. EVPI was calculated as 3.37 QALYs; therefore, the update metric is (2.41/3.37 =) 71.5%. This indicates that a model that updated the relative effect for treatment intensity and the relative effect for the new modality would have updated the parameters that covered 71.5% of the potential uncertainty. Even though all the other parameters have zero independent EVPPI, this does not reach 100% because the value of learning a set of parameters individually is not equal to learning about the full set simultaneously.

While this may be a useful first approximation, the conditional EVPPI algorithm is a more appropriate approach to ranking. Following the algorithm in Box 1 results in the ranking reported in Table 1.

**Table 1:** Rank of Parameter Importance and Update Metrics when Updating the Case Study Model with New Data

Input	Rank	Cumulative EVPPI	Marginal EVPPI	Update Metric
Relative effect for treatment intensity	1	0.36	0.36	10.7%
Relative effect for the new modality	2	2.41	2.05	71.5%
Baseline rate of local recurrence	3	3.47	1.06	102.9%

The analysis took less than 5 min. The results again show that only 2 parameters (the relative effect parameters) need to be updated to capture 71.5% of the total ex ante uncertainty in this model. Adding in the baseline rate of local recurrence has a large marginal EVPPI and results in the cumulative EVPPI approximating the EVPI. The marginal gains from updating the next most important parameter are very small by comparison. This suggests that efforts to update the model should be targeted at identifying new evidence for these 2 or 3 inputs. Note that because an efficient approximation method was used for EVPPI, the update metric is greater than 100% because the EVPPI is estimated with uncertainty and is associated with an upward bias.^14^

### Which Parameters Should Be Prioritized when Converting a Standard Decision Model to Take Account of Inequalities?

To estimate semielasticities, we fit a linear model to the PSA output of the cancer model case study. This linear model had an *R*^2^ of 88%, indicating a relatively linear model. The ranking and results are reported in Table 2 below.

**Table 2:** Rank of Parameter Importance and Adaptation Metrics when Converting the Case Study Model to a DCEA

Input	Rank	Semielasticity	Normalized Proportion	Standard Error	Adaptation Metric
Baseline rate of local recurrence	1	−0.0042	0.4626	≈0	46.3%
Utility with local recurrence	2	−0.0012	0.1352	0.0004	59.9%
Relative effect for treatment intensity	3	−0.0008	0.0897	≈0	68.8%
Relative effect for the new modality	4	−0.0006	0.0717	≈0	76.0%
Utility in well state	5	0.0005	0.0539	0.0004	81.8%
Baseline rate of metastatic disease	6	0.0004	0.0418	≈0	86.0%
Rate of metastatic disease following local recurrence	7	0.0003	0.0332	≈0	89.3%
Yearly costs with metastatic disease	8	0.0002	0.0253	≈0	91.8%
Mortality rate with metastatic disease	9	0.0002	0.0246	0.0002	94.3%
Yearly costs in well state	10	−0.0002	0.0184	≈0	96.2%
Yearly costs from second year after local recurrence	11	0.0001	0.0155	≈0	97.7%
Utility decrement from metastatic disease	12	0.0001	0.0068	≈0	98.4%
Costs in first year after local recurrence	13	0.0001	0.0064	≈0	99.0%
Probability of class 2a AE with A	14	≈0	0.0039	≈0	99.1%
Probability of class 2b AE with B	15	≈0	0.0032	≈0	99.4%
Probability of class 3 AE with A	16	≈0	0.0024	≈0	99.6%
Probability of class 2b AE with B	17	≈0	0.0023	≈0	99.7%
Probability of class 2a AE with B	18	≈0	0.0014	≈0	99.9%
Probability of class 3 AE with B	19	≈0	0.0011	≈0	100%
Additional costs in first year in well state	20	≈0	0.0004	≈0	100%
Probability of class 0 AE with B	21	≈0	0.0002	≈0	100%

AE, adverse events; DCEA, distributional cost-effectiveness analysis.

The analysis shows that 46.3% the total variation in OWSA results is from a single parameter: baseline rate of local recurrence. It also shows that adapting the top 10 input parameters will account for more than 90% of the total. Consequently, this analysis indicates that the marginal gains from adding parameters beyond rank 10 are extremely small. This means that even if there were large differences between groups in these parameters, including them in a DCEA is expected to have minimal impact on either INB for the total population or the magnitude of between-group differences in INB. Therefore, efforts should be targeted at identifying evidence on the top 10 input parameters in each of the equity relevant subgroups of interest, with more effort being spent on those higher in the ranking.

## Discussion

Parameters in a decision model can be updated with new information (updating) or adapted to reflect distributional information (adaptation). This article describes methods to prioritize parameters for model updating and adaptation. Metrics that can be reported alongside model updates or adaptations are also proposed. These metrics allow decision makers to understand the extent to which the important model parameters have been updated or adapted. We provided R code so that both analyses and quality metrics can be calculated quickly using only the results of a standard PSA.

Ideally, any update or adaptation should adjust every parameter in the model where new evidence is available; therefore, where quality scores are not 100%, analysts should justify why parameters have not been updated or adapted. As shown in the case studies, some parameters may be associated with very small marginal improvements in the metrics; if these require more substantial resource investments to adapt or update, then analysts may present the analysis here to justify their omission.

The metrics proposed measure the extent to which the most important parameters in a model have been updated or adapted. However, they do not quantify the quality or sample size of the data used to update or adapt the model. For parameters that are shown to be high priority, this analysis can be used to inform which parameters require systematic reviews where searching resources are limited.^18^ For parameters for which there is no suitable information in the literature, this analysis can also be used to justify more resource-intensive exercises to inform them such as evidence synthesis, analysis of individual patient data or expert elicitation.^3,6^ As a result of differences in intervention utilization across equity relevant groups, differences in INB from the intervention may be the main driver of differences in health between groups. Estimation of differences in utilization rates is a conceptually distinct issue and therefore research efforts cannot be prioritized using this method.

This article shows that ex ante quantification of parameter importance can use different methods whether updating or adapting decision models. For model updating, EVPPI is the relevant metric; for model adaptation, OWSA is the relevant and feasible approach. In this regard, these methods are not alternatives or substitutes for one another. They provide different information, relevant to different tasks. EVPPI and OWSA have been noted to correlate,^8^ and indeed, this correlation appears to hold in the case study above. This association is observed because VOI methods reflect 1) the degree of uncertainty about a parameter’s true value, 2) the degree to which the parameter influences costs and health consequences in the decision model, and 3) the extent of overall decision uncertainty. OWSA isolates the second of these aspects only. This specificity makes OWSA more suitable to prioritize parameters for distributional cost effectiveness analysis (DCEA). The degree of uncertainty about the expected value of a parameter, embodied in EVPPI, is not relevant to adapting models to DCEA. This is because the degree of uncertainty in the expected value of a parameter and the magnitude of differences in the value of a parameter across equity elevant groups may correlate but are conceptually distinct. It is worth highlighting the benefits of an EVPPI approach compared with an OWSA approach to prioritize parameters for updating. A comparison of Table 1 (ranking based on EVPPI) and Table 2 (ranking based on OWSA) demonstrates that the EVPPI analysis indicates that only 3 parameters would add value by being updated. In contrast, the OWSA does not provide a principled cutoff on the number of parameters to update. In general, it is not possible to know when to stop searching for parameters with OWSA, and if you stop too early, it is possible to miss out on a lot of value (if only 3 here, a key parameter from the EVPPI approach would be ignored) or if you stop too late you are expending resource for minimal, if any, gain.

Both the prioritization method and the metric for model updating are based on the standard VOI assumptions; therefore, they have the same limitations. First, they assume that the distributions on parameters from the existing model (i.e., priors) are valid (i.e., the uncertainty is appropriately characterized).^7,13,17^ To the extent to which the priors do not accurately reflect ex ante information on the parameters, the analysis will be inaccurate. Second, all VOI methods rely on parameterization of uncertainty of interest. In principle it is possible to parameterize structural uncertainty, although this may be challenging in practice.^15,20^ Therefore, structural uncertainty will most likely require qualitative consideration when prioritizing efforts for model updating.

The method proposed for prioritizing model adaptation for DCEA has a different set of limitations. These limitations primarily relate to model nonlinearity. The OWSA semielasticity does not quantify the degree of nonlinearity in the relationship between a parameter’s value and INB. Because failing to include heterogeneity in parameters with a greater degree of nonlinearity will result in incorrect estimates of expected outcomes, OWSA does not directly address the degree to which including between-group differences in particular parameters influence overall expected outcomes for the total population.^11^ An additional challenge of nonlinear models arises because the relationship between a change in a parameter and a change in INB is not linear. This means that the semielasticity may differ with larger versus smaller changes in the parameter and/or increases versus decreases. This makes interpretation more challenging because ex ante we do not have knowledge of the nature of the differences in the parameters across equity relevant groups. Further research is required to extend these models to the nonlinear case.

The limitations described above will affect the validity of the prioritization suggested by the methods in this article. However, as with all quantitative methods, we insist that the methods in this article should be one input into a wider decision-making process. Quantitative methods may provide useful insights, but the limitations of the underlying models need to be made explicit and taken account of.

There are a number of other extensions to the above methods that may be valuable. In both the update and adapt case studies, regression models were used to speed up and simplify the analysis. These models contain uncertain parameters. It is possible to propagate this uncertainty to present uncertainty intervals around estimates of rank and update/adaptation metrics. The EVPPI approach can easily generalize to multiple treatment options, but further work on the OWSA approach is required to extend it to more than 2 treatment alternatives.

A further extension is to use these methods to prioritize parameter searching across sets of decision models. Both methods quantify the importance of parameters in health terms; this means that after taking account of the incident population, the importance of parameters can be compared across models. This would allow parameter rankings and update/adaptation metrics to be calculated that follow the same principles in this article but include inputs from all decision models in the set.

## Supplemental Material

sj-docx-1-mdm-10.1177_0272989X241262037 – Supplemental material for Methods to Quantify the Importance of Parameters for Model Updating and Distributional Adaptation Supplemental material, sj-docx-1-mdm-10.1177_0272989X241262037 for Methods to Quantify the Importance of Parameters for Model Updating and Distributional Adaptation by David Glynn, Susan Griffin, Nils Gutacker and Simon Walker in Medical Decision Making
