# Supplementary material for: Methods to Quantify the Importance of Parameters for Model Updating and Distributional Adaptation
Source: Med Decis Making. 2024 Jul 26;44(7):802–10. doi: 10.1177/0272989X241262037 (PMC11490092; doi:10.1177/0272989X241262037)
Supplement: sj-docx-1-mdm-10.1177_0272989X241262037 – Supplemental material for Methods to Quantify the Importance of Parameters for Model Updating and Distributional Adaptation [file sj-docx-1-mdm-10.1177_0272989X241262037.docx]

# Appendix

## Case study model structure


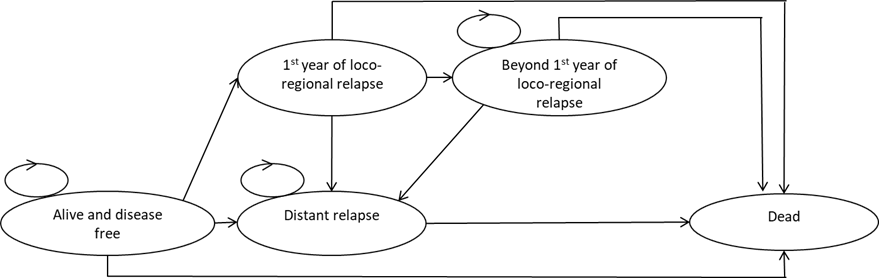


## Case study inputs

| **Input** | | **Mean** | **Standard Error** | **Distribution** |
| --- | --- | --- | --- | --- |
| Treatment costs | | | | |
|  | Treatment A | £2,314.50 | - | - |
|  | Treatment B | £953.60 | - | - |
| Population characteristics | | | | |
|  | Average age at beginning of model | 63 years | - | - |
| Acute side effects during treatment | | | | |
|  | Costs of treating adverse skin reactions for states: RTOG0, RTOG1, RTOG2a, RTOG2b, RTOG3 | £0, £0, £132, £132, £136 | - | - |
|  | Probability of RTOG states:  RTOG0, RTOG1, RTOG2a, RTOG2b, RTOG3 |  |  |  |
|  | Treatment A | 0%, 32%, 27.5%, 27.5%, 14% | - | Dirichlet |
|  | Treatment B | 6%, 62%, 13.5%, 13.5%, 6% | - | Dirichlet |
| Transition rate between states | | | | |
|  | Alive disease free to locoregional relapse:  Treatment A | 0.0022 | 0.0008 | Exponential |
|  | Alive disease free to distant relapse:  Treatment A | 0.0032 | 0.0009 | Exponential |
|  | Alive and disease free to death | Variable -dependant on age | - | - |
|  | Locoregional relapse to distant relapse | 0.0515 | 0.0045 | Exponential |
|  | Locoregional relapse to death | Variable - dependant on age | - | - |
|  | Distant relapse to death | 0.2196 | 0.5102 | Exponential |
| Relative treatment effect estimates | | | | |
|  | Alive disease free to locoregional relapse: hazard ratio for high intensity | 0.66 | 0.167 | Log normal |
|  | Alive disease free to locoregional relapse: hazard ratio for innovative modality | 0.88 | 0.510 | Log normal |
| Health care costs | | | | |
|  | Alive and disease free (annual) | £1,216 | £82 | Gamma |
|  | Additional costs of 1st year alive and disease free | £402 | £64 | Gamma |
|  | 1st year of locoregional relapse |  |  |  |
|  | Treatment costs | £4,241 | ±20% | Gamma |
|  | Supportive care costs | £2,995 | ±20% | Gamma |
|  | Beyond 1st year locoregional relapse (annual) | £2,139 | ±20% | Gamma |
|  | Distant relapse (annual) | £13,426 | ±20% | Gamma |
| Health related quality of life | | | | |
|  | Alive and disease free | 0.8302 | 0.0040 | Gamma |
|  | Locoregional relapse | 0.8302 | 0.0040 | Gamma |
|  | Decrement for distant relapse relative to alive and disease free | 0.3030 | 0.1550 | Gamma |
|  | Quality of life decrement with age | Variable -dependant on age | - | - |

RTOG = Radiation Therapy Oncology Group

## R code

### Overview

There is one function for model adaptation fn_adapt() and this takes two arguments:

- INB: The vector of incremental net benefits (in health or money terms) from the probabilistic sensitivity analysis (PSA). Used in both the update and adaptation analysis.
- PSA: The matrix of parameter values from the probabilistic sensitivity analysis (PSA). Used in both the update and adaptation analysis.

There are two functions for model updating: fn_update_independent () and fn_update_conditional() these carry out the “independent” and “conditional” EVPPI analysis respectively. As described in the paper, the conditional approach is theoretically more correct and it allows you to calculate the quality metric. However, it can take a longer time to compute and can rank a smaller number of variables.

Both functions require the following arguments:

- INB: described above
- PSA: described above
- incidence: the number of people impacted by the decision. This is an optional parameter and has no impact on ranking. Default is to report the expected value of partial perfect information (EVPPI) per person (i.e. incidence of 1).

The fn_update_conditional() takes two additional arguments

- interactions: if TRUE then uses tensor product when estimating EVPPI using the GAM, if FALSE then uses independent basis functions. The tensor product estimates the interactions between parameters but is a more complex function which may limit the number of parameters which can be ranked to four. See Strong et al 2014 for more details.
- max_params: the maximum number of parameters which will be ranked. Note that ranking more than 4 parameters with interactions could cause problems for the GAM model. See Strong et al 2014 for more details.

### Code

### Introduction #####

# Created by David Glynn 9/3/23

# This code creates three end user functions (fn_adapt, fn_update_independent and fn_update_conditional)

# and four support functions, called by the user functions (fn_clean_PSA, fn_EVPI, evpi.gam.SE.bias, fn_EVPPI_GAM)

# Code from Mark Strong used to create evpi.gam.SE.bias function.

# required packages

library(MASS) # sample mvn distribution in calculating standard error of EVPPI

library(mgcv) # GAM function used to calculate EVPPI

### FUNCTION: fn_clean_PSA #########

# cleans up the PSA dataset (remove variables with no variation and NAs)

# INPUTS:

# PSA = The matrix of parameter values from the probabilistic sensitivity analysis (PSA)

# OUTPUTS:

# PSA_clean = PSA matrix without variables with no variation and NAs

fn_clean_PSA <- function(PSA){

# remove variables with no variation or containing NAs

nonzero_variation <- as.numeric(ifelse(apply(PSA, 2, function(x) sd(x)) == 0, 0, 1))

# assign 0 to NA values

nonzero_variation[is.na(nonzero_variation)] <- 0

# create non zero vector

PSA_clean <- PSA[,1:dim(PSA)[2]*nonzero_variation]

return(PSA_clean)

}

### FUNCTION: fn_adapt ####

# Prioritizing parameters for distributional analysis

# INPUTS:

# INB = The vector of incremental net benefits (in health or money terms) from the probabilistic sensitivity analysis (PSA)

# PSA = The matrix of parameter values from the probabilistic sensitivity analysis (PSA)

# OUTPUTS:

# output = a list with two components:

# output$adapt_table = a data frame with each parameter ranked in terms of importance for adaptation

# output$adapt_model = the linear model used to rank parameters for importance

fn_adapt <- function(

INB = INB,

PSA = PSA){

## clean PSA inputs (remove variables with no variation or containing NAs)

PSA <- fn_clean_PSA(PSA)

## calculate E(x_j) for every input

v_mean_x_j <- colMeans(PSA)

### semi elasticities

## multiple regression with y = INB = percentage change in x

## convert x values to % change from mean, 1 = 1% higher than mean, -2 = 2% lower than mean

PSA_prec <- as.data.frame(apply(PSA, 2, function(x) ((x - mean(x))/mean(x))*100 ))

## fit linear model on incremental QALYs with all variables

lm_INB <- lm(INB ~ ., data = PSA_prec)

# summary(lm_INB)

# vector of a_i coefficients for all x (coefficients = 1% elasticities)

v_a_i <- lm_INB$coefficients[-1]

# summary(lm_INB)$coefficients[-1, 2]

# vector of p-values for all x

v_p_value <- summary(lm_INB)$coefficients[-1,4]

# vector of SE for x_j

v_se <- summary(lm_INB)$coefficients[-1, 2]

## calculate change in y from 1% increase in each variable

v_delta_y <- v_a_i

# remove rows with nas ?

v_delta_y<-v_delta_y[!is.na(v_delta_y)]

# standardise to 100%

abs_v_delta_y <- abs(v_delta_y)

normalised_values <- abs_v_delta_y/sum(abs_v_delta_y)

## create table of parameters ordered from most to least important,

# named table with absolute deviations, p-values and normalised proportions

adapt_table <- data.frame(

Delta_p = v_delta_y,

Pvalue = v_p_value,

SE = v_se,

Proportion = normalised_values

)

# sort by size of normalised value

adapt_table <- adapt_table[order(-adapt_table$Proportion),]

# add cumulative sum

adapt_table$Cumulative <- cumsum(adapt_table$Proportion)

# add marginal proportion

adapt_table$Marginal <- adapt_table$Cumulative - c(0, head(adapt_table$Cumulative, -1))

# add in ranking

adapt_table$Rank <- 1:dim(adapt_table)[1]

# add in quality score (cululative as percentage)

adapt_table$Quality <- round(adapt_table$Cumulative*100, 1)

# include names of columns

colnames(adapt_table) <- c("Delta +1%", "P-value", "SE" , "Proportion", "Cumulative", "Marginal", "Rank", "Quality")

# reorder columns to put rank at start

adapt_table <- adapt_table[, c("Rank", "Delta +1%", "Proportion", "SE" , "Quality", "Cumulative", "Marginal", "P-value")]

# define degree of rounding

adapt_table <- round(adapt_table, 4)

## outputs from the function

output <- list(

adapt_table = adapt_table,

adapt_model = lm_INB

)

return(output)

}

### FUNCTION: fn_EVPI ##########

# INPUTS:

# INB = The vector of incremental net benefits (in health or money terms) from the probabilistic sensitivity analysis (PSA)

# incidence = the number of people impacted by the decision. Default is EVPPI per person (i.e. incidence of 1)

# OUTPUTS:

# output = a list with three components:

# output$EVPI = EVPI for the incident population

# output$Probability_cost_effective = the probability of each of the treatment options being cost effective

fn_EVPI <- function(INB = INB,

incidence = 1

){

## calculate EVPI

# create matrix of payoffs for current practice (column 1) and new treatment (column 2)

INB_t <- cbind(0, INB)

# expected outcome with each treatment

ENB_t <- apply(INB_t , 2, mean)

# Best outcome with current information

NB_EVTCI = max( ENB_t , na.rm = TRUE)

# Expected value of treating with perfect information

NB_VTPI <- apply(INB_t , 1, max, na.rm = TRUE) #so I can check convergence

NB_EVTPI <- mean( NB_VTPI )

NB_EVPI <- NB_EVTPI - NB_EVTCI

## calculate probability of being optimal treatment

Probability_cost_effective <- apply(INB_t , 2, function(x) sum(x==NB_VTPI , na.rm = TRUE))/length(NB_VTPI)

names(Probability_cost_effective) <- c("Current practice", "New treatment")

## outputs from the function

output <- list(

EVPI = NB_EVPI*incidence,

Probability_cost_effective = Probability_cost_effective

)

return(output)

}

### FUNCTION: evpi.gam.SE.bias ##########

# Code developed by Mark Strong https://www.sheffield.ac.uk/scharr/people/staff/mark-strong

# INPUTS:

# INB = The vector of incremental net benefits (in health or money terms) from the probabilistic sensitivity analysis (PSA)

# PSA = The matrix of parameter values from the probabilistic sensitivity analysis (PSA)

# regression.model = regression model functional form for GAM used to calculate EVPPI

# S = iterations used in calculating standard error and upward bias

# OUTPUTS:

# list called partial.evpi with three elements

# partial.evpi$partial.evpi = EVPPI

# partial.evpi$SE = standard error for the EVPPI

# partial.evpi$upward.bias = an estimate of upward bias for the EVPPI

evpi.gam.SE.bias <- function(INB, PSA, regression.model, S = 1000) {

# create matrix of payoffs for current and new intervention

NB <- cbind(0, INB)

D <- ncol(NB)

N <- nrow(NB)

g.hat <- beta.hat <- Xstar <- V <- tilde.g <- vector("list", D)

g.hat[[1]] <- rep(0, N)

for (d in 2:D) {

# print(paste("estimating g.hat for incremental NB for option", d, "versus 1"))

f <- update(formula(NB[, d] ~ .), formula(paste(".~", regression.model)))

model <- gam(f, data = data.frame(PSA))

g.hat[[d]] <- model$fitted

beta.hat[[d]] <- model$coef

Xstar[[d]] <- predict(model, type = "lpmatrix")

V[[d]] <- model$Vp

}

perfect.info <- mean(do.call(pmax, g.hat))

baseline <- max(unlist(lapply(g.hat, mean)))

partial.evpi <- perfect.info - baseline ## estimate EVPI

rm(g.hat)

gc()

# print("computing standard error and upward bias via Monte Carlo")

for (d in 2:D) {

sampled.coef <- mvrnorm(S, beta.hat[[d]], V[[d]])

tilde.g[[d]] <- sampled.coef %*% t(Xstar[[d]])

}

tilde.g[[1]] <- matrix(0, nrow = S, ncol = N)

rm(V, beta.hat, Xstar, sampled.coef)

gc()

sampled.perfect.info <- rowMeans(do.call(pmax, tilde.g))

sampled.baseline <- do.call(pmax, lapply(tilde.g, rowMeans))

rm(tilde.g)

gc()

sampled.partial.evpi <- sampled.perfect.info - sampled.baseline

SE <- sd(sampled.partial.evpi)

upward.bias <- mean(sampled.partial.evpi) - partial.evpi

return(list(partial.evpi = partial.evpi, SE = SE, upward.bias = upward.bias))

}

### FUNCTION: fn_EVPPI_GAM ########

# wrap Mark's GAM function

# take inputs and calculates EVPPI for the parameter(s) indexed

# INPUTS:

# INB = The vector of incremental net benefits (in health or money terms) from the probabilistic sensitivity analysis (PSA)

# PSA = The matrix of parameter values from the probabilistic sensitivity analysis (PSA)

# sets = Index number of variables to calculate EVPPI for

# interactions = if TRUE then uses tensor product when estimating GAM, if FALSE then just uses independent basis functions

# OUTPUTS:

# output which is a list with three elements

# output$partial.evpi = EVPPI for the inputs specified

# output$SE = standard error for the EVPPI for the inputs specified

# output$upward.bias = upward bias for the EVPPI for the inputs specified

fn_EVPPI_GAM <- function(INB, PSA, sets, interactions){

# names of variables to calcualte EVPPI for

variables <- names(PSA)[sets]

# construct right hand side of model input

if(interactions == TRUE){

if(length(sets) == 1){

regression.model <- paste0("s(", paste(variables, collapse = " , "), ")")

} else {

# If there are more than three parameters that are expected to interact set a maximum basis dimension of 4

# This will avoid the model trying to estimate too many coefficients "te(x1,x2,x3,x4,k=4)"

regression.model <- paste0("te(", paste(variables, collapse = " , "), ", k=4)" )

}

} else {

regression.model <- paste0("s(", paste(variables, collapse = " , "), ")")

}

# calculate EVPPI

output <- evpi.gam.SE.bias(INB = INB,

PSA = PSA,

regression.model = regression.model)

return(output)

}

### FUNCTION: fn_update_independent #############

# Prioritizing parameters for updating (based on independent EVPPI values)

# INPUTS:

# INB = The vector of incremental net benefits (in health or money terms) from the probabilistic sensitivity analysis (PSA)

# PSA = The matrix of parameter values from the probabilistic sensitivity analysis (PSA)

# incidence = the number of people impacted by the decision. Default is EVPPI per person (i.e. incidence of 1)

# interactions = if TRUE then uses tensor product when estimating GAM, if FALSE then just uses independent basis functions

# OUTPUTS:

# output = a list with one component:

# output$update_table = a dataframe with each parameter ranked in terms of importance for updating

fn_update_independent <- function(

INB,

PSA,

incidence = 1

){

## clean PSA inputs (remove variables with no variation or containing NAs)

PSA <- fn_clean_PSA(PSA)

## initialise vectors

# vector holding EVPPI results

v_EVPPI <- rep(NA, dim(PSA)[2])

# vector holding SE for EVPPI results

v_EVPPI_SE <- rep(NA, dim(PSA)[2])

# vector holding bias estimates for EVPPI results

v_EVPPI_bias <- rep(NA, dim(PSA)[2])

# loop through variables and calculate EVPPI for each parameter independent of the other parameters

for(i in 1:dim(PSA)[2]){

output <- fn_EVPPI_GAM(INB = INB,

PSA = PSA,

sets = i,

interactions = FALSE)

v_EVPPI[i] <- output$partial.evpi

v_EVPPI_SE[i] <- output$SE

v_EVPPI_bias[i] <- output$upward.bias

print(paste("EVPPI calculated for", i, "out of", dim(PSA)[2]))

}

## create table of EVPPI values ordered from most to least important,

# named table with EVPPI p-values and normalized proportions

update_table <- data.frame(

EVPPI = v_EVPPI*incidence,

SE = v_EVPPI_SE*incidence,

Bias = v_EVPPI_bias*incidence,

Proportion = v_EVPPI/sum(v_EVPPI)

)

# add parameter names to rows of table

rownames(update_table) <- names(PSA)

# sort by size of normalized value

update_table <- update_table[order(-update_table$Proportion),]

# add cumulative sum

update_table$Cumulative <- cumsum(update_table$Proportion)

# add marginal proportion

update_table$Marginal <- update_table$Cumulative - c(0, head(update_table$Cumulative, -1))

# add in ranking

update_table$Rank <- 1:dim(update_table)[1]

# reorder columns to put rank at start

update_table <- update_table[, c("Rank", "EVPPI", "Proportion", "SE" , "Cumulative", "Marginal", "Bias")]

## outputs from the function

output <- list(

update_table = update_table

)

return(output)

}

### FUNCTION: fn_update_conditional #############

# Prioritizing parameters for updating (based on conditional EVPPI values)

# INPUTS:

# INB = The vector of incremental net benefits (in health or money terms) from the probabilistic sensitivity analysis (PSA)

# PSA = The matrix of parameter values from the probabilistic sensitivity analysis (PSA)

# incidence = the number of people impacted by the decision. Default is EVPPI per person (i.e. incidence of 1)

# interactions = if TRUE then uses tensor product when estimating GAM, if FALSE then just uses independent basis functions

# max_params = the maximum number of parameters to rank (ranking more than 4 with interactions could cause problems for the model)

# OUTPUTS:

# output = a list with one component:

# output$update_table = a data frame with each parameter ranked in terms of importance for updating

fn_update_conditional <- function(

INB,

PSA,

incidence = 1,

interactions = TRUE,

max_params = 4

){

## clean PSA inputs (remove variables with no variation or containing NAs)

PSA <- fn_clean_PSA(PSA)

# calculate EVPI (for use in calculating quality metric)

EVPI <- fn_EVPI(INB = INB,

incidence = incidence)$EVPI

# number of parameters in total

n_params <- dim(PSA)[2]

# intialise data structure to hold main results

update_table <- data.frame(name = rep(NA, n_params),

rank = 1:n_params,

cumulative_EVPPI = rep(NA, n_params),

SE = rep(NA, n_params),

Up_bias = rep(NA, n_params),

Update_metric = rep(NA, n_params))

# captures time taken for each round

round_time <- rep(NA, n_params - 1)

# vector containing the index number of the top ranked parameters (begins empty)

top_params <- NULL

# vector containing the index number of yet to be ranked parameters (begins full)

remaining_params <- 1:n_params

# in each round EVPPI is calculated and the top parameter is identified

# after each round the top parameters in the previous round are added to the EVPPI equation

# continue until all parameters ranked

for(r in 1:min(max_params, n_params - 1)){

# start clock for round r

start.time_r <- Sys.time()

# vector to contain results in round r

v_EVPPI <- rep(NA, n_params)

v_EVPPI_SE <- rep(NA, n_params)

v_EVPPI_bias <- rep(NA, n_params)

for(i in remaining_params){

output <- fn_EVPPI_GAM(INB = INB,

PSA = PSA,

sets = c(top_params, i),

interactions = interactions)

v_EVPPI[i] <- output$partial.evpi

v_EVPPI_SE[i] <- output$SE

v_EVPPI_bias[i] <- output$upward.bias

}

# index of element which has maximum value and is not an outlier

top_param_r <- which.max(v_EVPPI)

# identify index of top ranked parameter in round r

# record resutts in table

update_table[r, 1] <- names(PSA)[top_param_r]

update_table[r, 3] <- v_EVPPI[top_param_r]*incidence

update_table[r, 4] <- v_EVPPI_SE[top_param_r]*incidence

update_table[r, 5] <- v_EVPPI_bias[top_param_r]*incidence

update_table[r, 6] <- ((v_EVPPI[top_param_r]*incidence)/EVPI)*100

# update top parameters and remaining parameters for next round

top_params <- c(top_params, top_param_r)

remaining_params <- remaining_params[! remaining_params %in% top_param_r]

# end clock round r

end.time_r <- Sys.time()

time.taken_r <- end.time_r - start.time_r

print(time.taken_r)

round_time[r] <- time.taken_r

# progress report

print(paste(length(top_params), "of", max_params, "ranked, table", round(length(top_params)/max_params*100), "% complete" ))

# stop the loop if the estimated EVPPI is greater than the total EVPI

if(v_EVPPI[top_param_r]*incidence > EVPI) break

}

# clean up table

update_table <- update_table[1:r,]

## outputs from the function

output <- list(

update_table = update_table

)

return(output)

}
